# High-Accuracy Brain-Machine Interfaces Using Feedback Information

**DOI:** 10.1371/journal.pone.0103539

**Published:** 2014-07-30

**Authors:** Hong Gi Yeom, June Sic Kim, Chun Kee Chung

**Affiliations:** 1 Interdisciplinary Program in Neuroscience, Seoul National University, Seoul, Republic of Korea; 2 MEG center, Seoul National University Hospital, Seoul, Republic of Korea; 3 Department of Neurosurgery, Seoul National University Hospital, Seoul, Republic of Korea; 4 Sensory Organ Research Institute, Seoul National University, Seoul, Republic of Korea; 5 Department of Brain and Cognitive Sciences, Seoul National University College of Natural Sciences, Seoul, Republic of Korea; McGill University, Canada

## Abstract

Sensory feedback is very important for movement control. However, feedback information has not been directly used to update movement prediction model in the previous BMI studies, although the closed-loop BMI system provides the visual feedback to users. Here, we propose a BMI framework combining image processing as the feedback information with a novel prediction method. The feedback-prediction algorithm (FPA) generates feedback information from the positions of objects and modifies movement prediction according to the information. The FPA predicts a target among objects based on the movement direction predicted from the neural activity. After the target selection, the FPA modifies the predicted direction toward the target and modulates the magnitude of the predicted vector to easily reach the target. The FPA repeats the modification in every prediction time points. To evaluate the improvements of prediction accuracy provided by the feedback, we compared the prediction performances with feedback (FPA) and without feedback. We demonstrated that accuracy of movement prediction can be considerably improved by the FPA combining feedback information. The accuracy of the movement prediction was significantly improved for all subjects (*P*<0.001) and 32.1% of the mean error was reduced. The BMI performance will be improved by combining feedback information and it will promote the development of a practical BMI system.

## Introduction

The brain–machine interface (BMI) is a promising technology that will help disabled people to interact with the external world. Many BMI studies have been performed over the past few decades [1]–[5], the results of which have made it possible for a monkey or human to control a robotic arm through neural activity to eat or drink [6]–[8]. However, the accuracy of controlling a robotic arm is quite low. For example, in a recent study, success rates were 20.8%–62.2% for reaching and grasping movements [7].

Although the robotic arm approximately reached a target, grasping movements were often failed because the robotic arm did not exactly reach an object. The movement prediction inaccuracy is a critical barrier to practical application [9].

Such inaccuracy problem could be overcome by using feedback information. Movement control is achieved from not only motor commands but also sensory feedback [10]. Animals and humans compensate their movement errors by the feedback such as the position information obtained from proprioception and vision. Therefore, feedback information should also be considered in BMI system for high-accuracy. However, feedback information has not been directly used to update movement prediction model in the previous BMI studies, although the closed-loop BMI system provides the visual feedback to users. Therefore, efforts and times for adaptation are required to BMI users.

**Figure 1:**
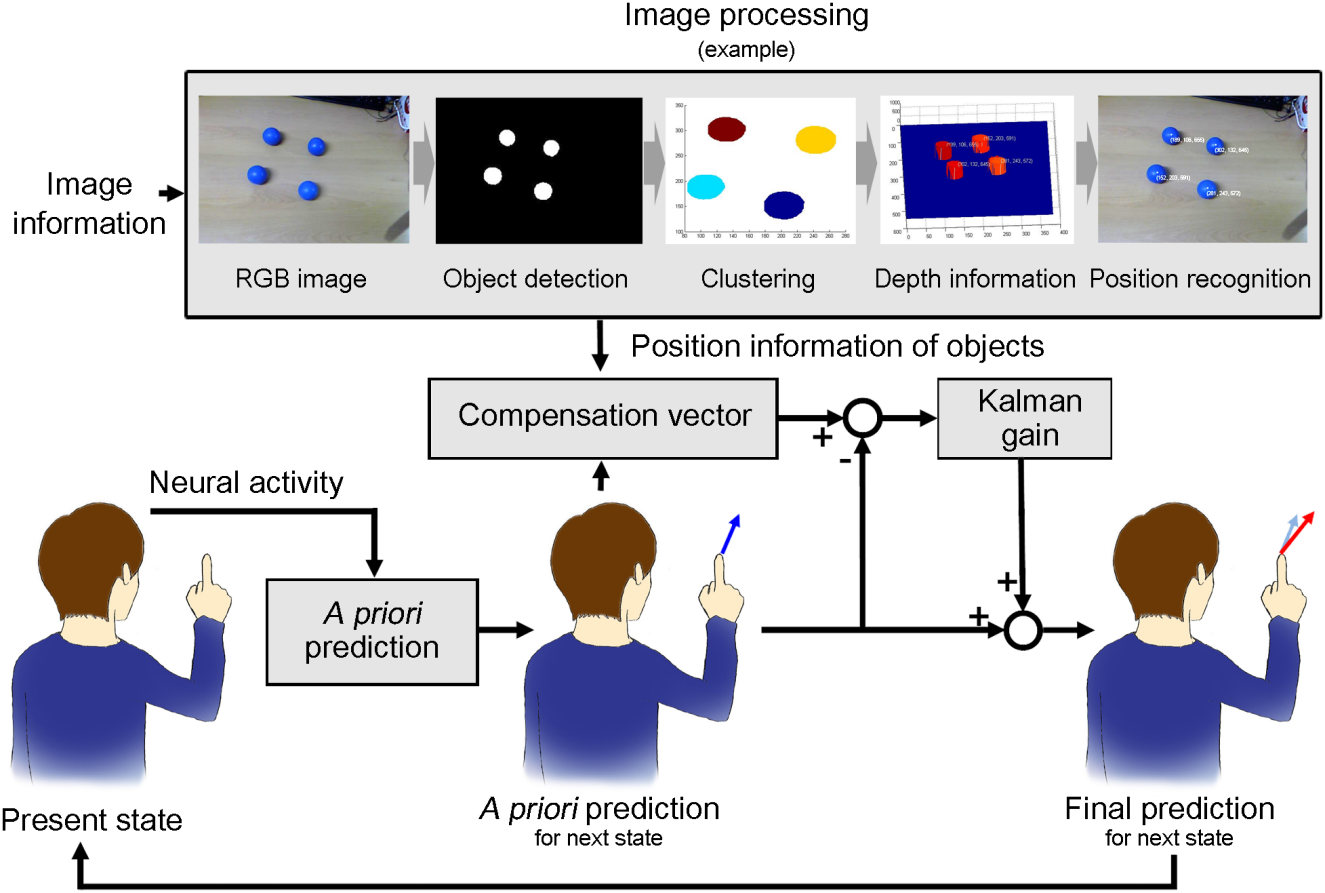
A BMI framework combining image processing. The BMI framework receives image information through external device. Position information of objects are calculated from the image information by image processing. The proposed FPA algorithm generates a compensation vector based on the position information and *A priori* prediction. The purpose of the compensation vector is to rotate the prediction vector toward the predicted target and magnify the predicted vector to easily reach the target. The FPA predicts the movement and compensates using the position information recursively.

Unfortunately, it is difficult to extract the sensory feedback from the neural activity. Instead, we can obtain useful information by adding a stereo camera to the BMI system. For example, the positions of objects can be calculated from an image recorded by an external camera and movement prediction can be compensated toward the object position as a movement goal. The positions of objects can be easily calculated from the image by the image segmentation method, which is a conventional technique (Fig. 1; see [11]).

**Figure 2:**
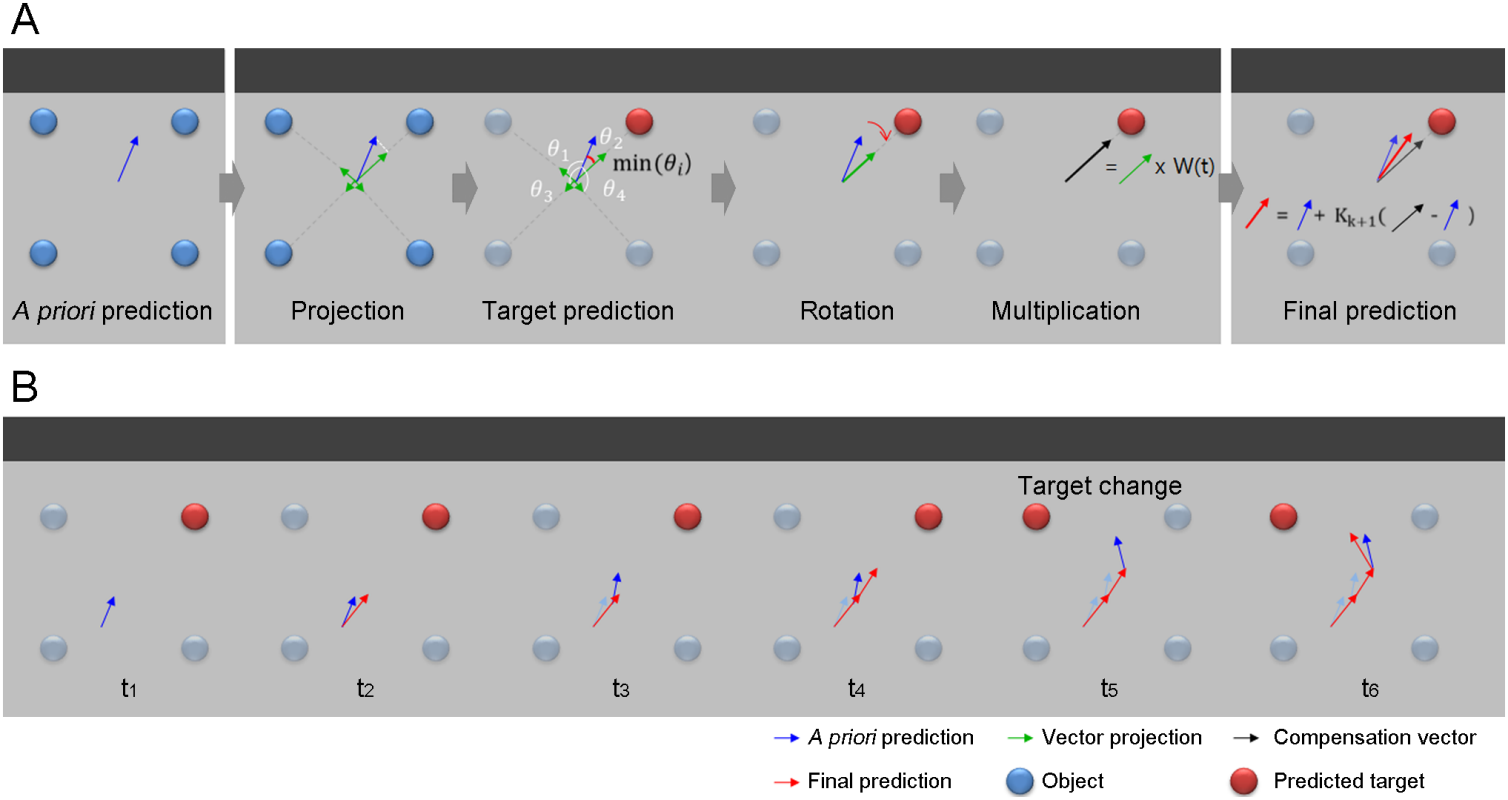
Principle of the FPA. (**A**) Three steps of the FPA. Step 1, the next movement state is *a priori* predicted from the present state and the neural activity. Step 2, the *a priori* predicted vector is projected onto the vectors directed from the present position to each object (green arrows are vector projections). One of the objects is predicted as the target which has the minimal angle between the predicted vector and vector projections (the red sphere). The predicted vector is rotated toward the target and multiplied by a weight value (black arrow) to magnify the predicted vector to easily reach the target. Step 3, the final prediction vector (red arrow) is determined from *a priori* prediction and the compensation vector. (**B**) Prediction example while changing a target. The example shows how a target and final prediction can be changed according to the direction of *a prior* prediction.

Here, we propose a BMI framework combining image processing with a novel prediction method, the feedback-prediction algorithm (FPA) that generates feedback information from the positions of objects and modifies movement prediction with the feedback (Figs. 1, and 2). The FPA predicts a target among objects based on the movement direction predicted from the neural activity. After the target selection, the FPA modifies the predicted direction toward the target and modulates the magnitude of the predicted vector to easily reach the target (Fig. 2A). The FPA repeats the modification in every prediction time points. To evaluate the performance improvements provided by the feedback, we predicted 3-dimensional reaching movements from MEG signals in both cases with feedback (FPA) and without feedback and then compared the prediction accuracy.

## Materials and Methods

### Ethics Statement

Prior to the study, all subjects submitted written informed consent for participating in the study. This study was approved by the Institutional Review Board of the Seoul National University Hospital (1105-095-363).

### Data acquisition and signal processing

To decode the movements, we used the identical features as described in our previous study and the present datasets were used previously [12]. Briefly, 9 healthy subjects participated in the experiment (age: 19–37 years; five men and four women). The MEG signals were measured using a 306-channel whole-head MEG system (VectorView TM, Elekta Neuromag Oy, Helsinki, Finland) during arm movements in 3D space. A three-axis accelerometer (KXM52, Kionix, NY, USA) was used to record movement trajectories. The accelerometer was placed on the index finger and the sensor signals were simultaneously recorded with the MEG signals. To guide three-dimensional reaching movements, stereographic images were presented on a screen. At the beginning of the experiment, a sphere was presented on the center of the screen for 4 s and a target sphere with a stick connecting it to the center sphere appeared on one corner for 1 s. The target sphere was presented randomly on one of the four corners (upper-left, upper-right, bottom-left, and bottom-right). During this time, the subject was instructed to use his/her arm and to reach his/her index finger from the center to the target and come back to the center as fast as possible along the stick line (center-out-center paradigm). For each subject, 60 trials for each direction were measured.

For the movement prediction, we selected 68 gradiometer channels on motor-related areas based on power spectrum analysis. The MEG signals were band-pass filtered at 0.5–8 Hz, and downsampled to 50 Hz. Eleven data points preceding the current data point were used as features for predicting velocity. The movement velocities of x, y, and z were predicted from the regression method without feedback and with feedback (FPA). After the movement velocity prediction, the movement trajectories were calculated by integrating the predicted velocities.

The MEG and accelerometer data are located in Data S1.

Because the stereographic images were presented instead of real objects in our experiment, we assumed that object positions are equal to the mean position of the end points of real movement trajectory instead of the real image processing.

### Feedback-prediction algorithm (FPA)

In previous BMI studies using a Kalman filter, the next state was usually predicted from the present state and the prediction was compensated based on the neural signals [13]–[15]. Therefore, the method ensures that the prediction maintains the direction of the previous movements and it diminishes variation of prediction. This approach can be beneficial in the case of the prediction for smooth movements. However, the method may hinder the prediction of movement with rapid change. In robotics, the system generally estimates the next state from the present state with input signals and compensates the prediction based on measurement value, when measurement is possible [16]. Therefore, it is more reasonable to predict the next state from the present state with neural activity and compensate the prediction by the measurement such as with the proposed FPA.

The FPA is a recursive prediction algorithm consisting of three steps: 1) *a prior* prediction, 2) generation of a compensation vector, and 3) final prediction. In the *a prior* prediction step, the next movement state was predicted by the multiple linear regression (MLR) from the previous movement state and the MEG signals. The *a prior* prediction method corresponds to the general prediction method used in various BMI studies [5], [6], [12], [17]–[20]. In the generation step of a compensation vector, a target is predicted among the objects based on the direction of the *a prior* prediction vector. After the target selection, a new vector directing target from a present position is created. The magnitude of the vector is modified based on the probability that the predicted target is a real target by multiplying a weight value. This is a compensation vector which is used as feedback information. The weight value helps the movement prediction easily reach the target. Lastly, the final prediction is determined by adding the Kalman gain-multiplied error (the difference between the *a prior* prediction and the compensation vector), to the *a prior* prediction. The process of the FPA is as follows (Fig. 2A).

#### Step 1. *A priori* prediction

In the first step, the next movement state was predicted from the previous movement state and the MEG signals. The relation between states, inputs, and measurements can be described with the state equation and output equation as follows:
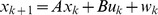


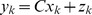
where 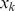 is the state matrix (position) at time k; 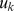 indicates the MEG signal matrix; 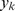 is the measurement matrix which corresponds to a compensation vector; 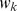 describes the noise matrix and 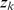 is the measurement error matrix; A, B, and C are the coefficient matrices. In our study, we assumed that the matrix A, C is an identity matrix. B was calculated using the multiple linear regression.

To predict next k+1 state at time k, the FPA *a priori* predicts the next state of movements from the present state and neural activity as follows:
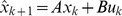


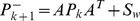
where 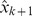 describes the *a priori* predicted next state and 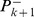 is an *a priori* prediction error covariance and where 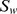 is a covariance matrix of system noise. We defined 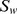 as follows:



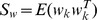



#### Step 2. Generation of a compensation vector

In the second step, a target is predicted among the objects and a compensation vector is generated. To predict a target, the *a priori* predicted vector is projected onto the vectors directed from the present position to each object.
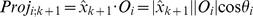
where 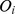 is a unit vector pointing to each object *i*; 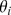 is an angle between 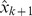 and 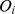. The length of the vector projection represents the degree of similarity of the predicted vector to the vector pointing to each object because the length of the vector projection is inversely proportional to the angle 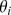 between the predicted vector and the vector directing the object. Therefore, an object corresponding to the maximal vector projection is predicted as the target as follows:



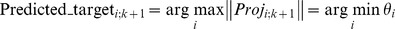
where 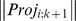 is a Euclidean distance of the vector projection 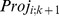. Because the target is predicted in every FPA process based on the neural activity, the subject can change his/her movement goal at any time (Fig. 2B).

After the target selection, the vector projection pointing to the target is multiplied by a weight value W(t). The purpose of multiplying the weight value is to help to easily reach the target. The weight value W(t) was calculated by dividing the length of projection vector pointing to the target with the mean length of the projection vectors as follows:
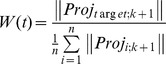


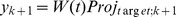
where 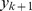 is the compensation vector. The weight W(t) was restricted to 2 to prevent overweight. We selected the appropriate restriction value by the experiment.

#### Step 3. Final prediction

In the final step, the final prediction vector is determined using the *a priori* prediction vector and a compensation vector calculated from the first and second steps. The *a priori* prediction 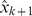 is compensated with the compensation vector 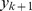 as follows:
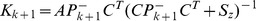


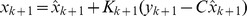


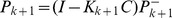
where 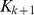 is called the Kalman gain and 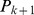 is a *posteriori* prediction error covariance; the −1 superscript indicates the matrix inversion, the T superscript represents the matrix transposition; and 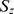 is a covariance matrix of the measurement error. We defined the 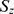 as follows:



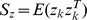



We assumed that the 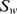 and the 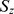 were same and they were identity matrices in our study.

### Evaluation of the performance

We compared the performance in cases with feedback (FPA) and without feedback. To evaluate the performance, we assessed the closeness of the end points of the predicted trajectory to the target. We defined the error by the distances from the end point of the predicted trajectory to the target position, which was divided by the distance from the origin to the target position to normalize the error. In addition, movement error (ME) and movement variability (MV) were calculated [21]. ME represents an average distance of the predicted trajectory from the task axis. ME means how much a predicted trajectory is far from the ideal straight line. MV measures the standard deviation between a predicted trajectory and the task axis. MV depicts the variation of the predicted trajectory. For statistical analysis, we applied a paired-samples t-test to the errors in cases with feedback (FPA) and without feedback using SPSS, version 13.0 (SPSS, Chicago, IL).

## Results

The results of the evaluation demonstrate that the end points of the trajectory predicted with the feedback were closer to the target and also more focused on the target than the end points predicted without feedback, because the magnitude and the direction of the predicted movement with feedback were modulated toward the target using the feedback information (Figs 1 and 2 and Video S1).

**Figure 3:**
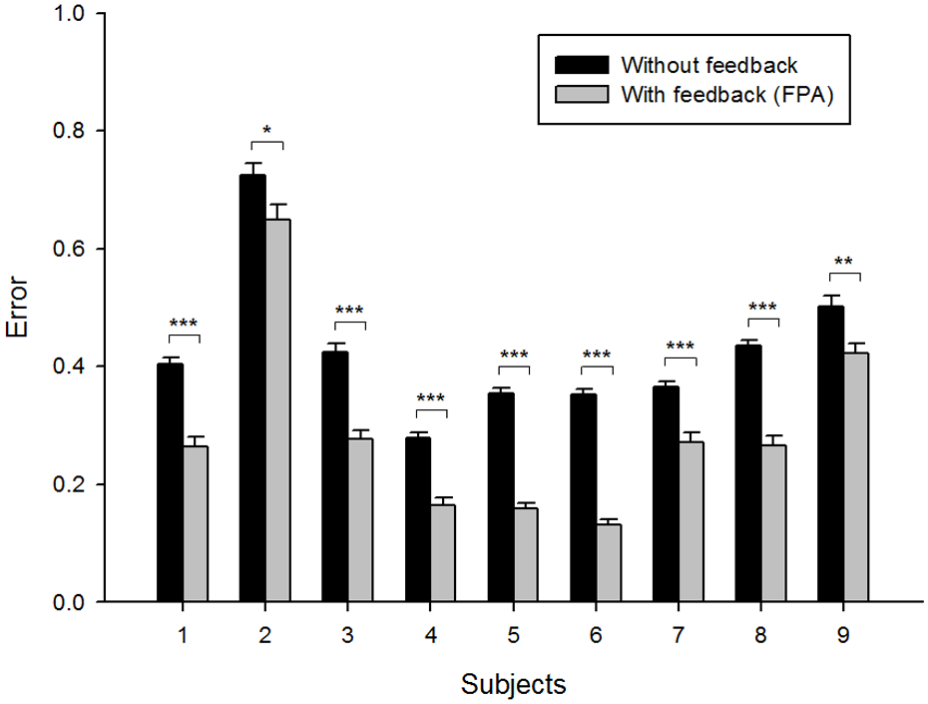
Error bar with standard error for each subject. Black bars illustrate errors in a case without feedback and gray bars represent errors in a case with feedback (FPA). *P = 0.021, **P = 0.002, ***P<0.001.

The paired-samples t-test showed a significant group difference between errors in cases with and without feedback (*P*<0.001), implying that the performance of the movement prediction was significantly improved by feedback (FPA). The mean error declined from 0.427±0.238 to 0.290±0.288 (mean ± SD) with feedback, corresponding to an error reduction of 32.1%. Because the reaching target was the virtual sphere, the variation of the real movements from the target center (error) was 0.178±0.131. Based on the consideration of the real movement variation, the error of the FPA is considerably low. Fig. 3 illustrates the error bar and standard error in cases with feedback (gray) and without feedback (black) for each subject. We also evaluated the individual difference between errors in cases with feedback and without feedback by the paired-samples t-test. The *p*-values of most subjects were under 0.001 (*p* = 0.021 and *p* = 0.002 for subject 2 and subject 9, respectively).

Moreover, ME and MV were significantly decreased by feedback (*P*<0.001 and *P*<0.05, respectively). The mean ME without feedback was 0.1146±0.0722 and the mean ME with feedback was 0.0811±0.0925. The mean MV without feedback was 0.0724±0.0512 and the mean MV with feedback was 0.0698±0.0850. The results represent that predicted trajectories were closed to the optimal path and the variations of the predicted trajectories were reduced by feedback.

Note that the prediction results without feedback already showed high performance (mean *r*>0.7) as described in our recent study [12]. Nevertheless, performance was significantly improved by combining the feedback information generated from the positions of objects.

**Figure 4:**
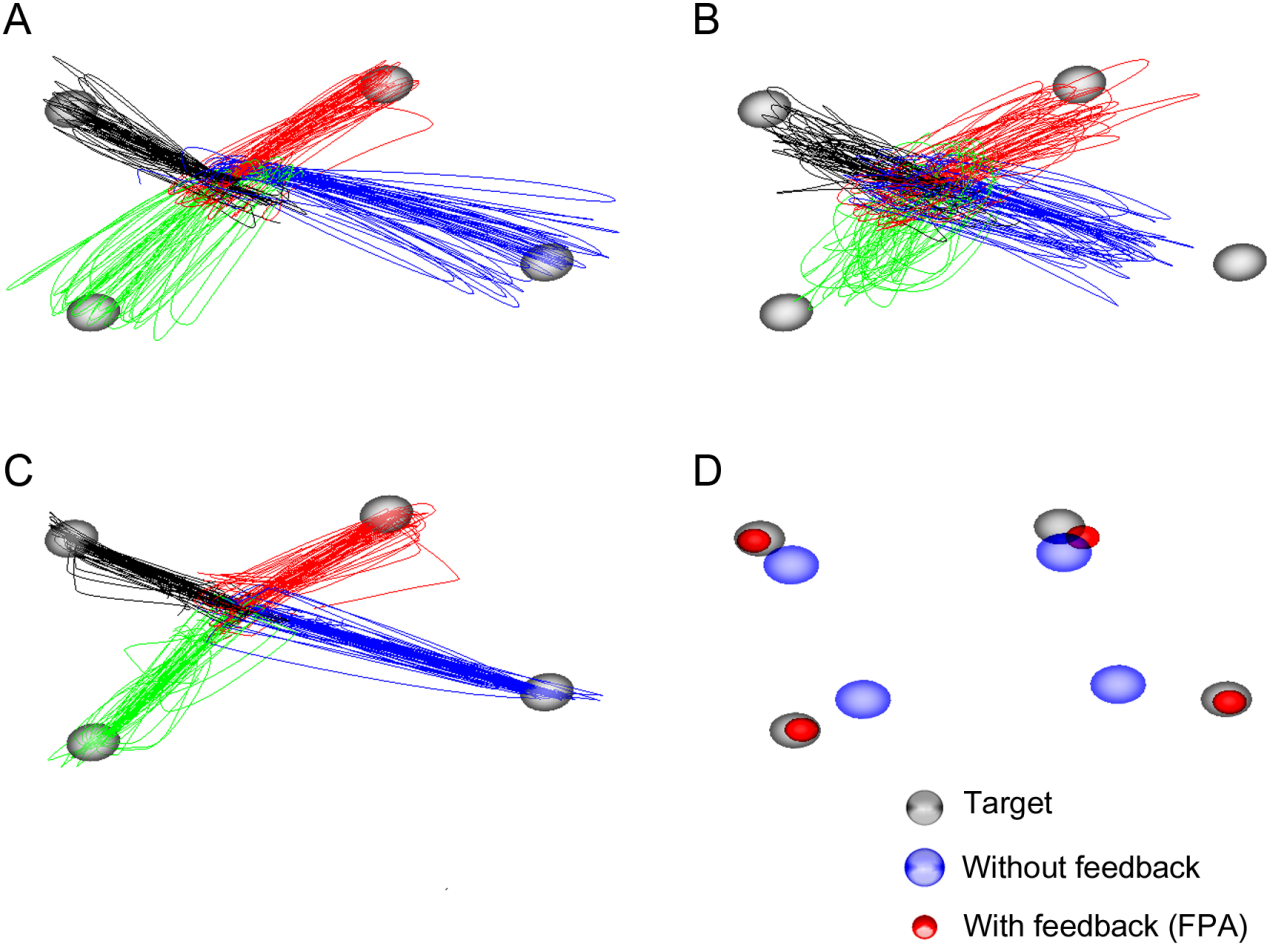
Examples of the movement prediction in 3D space in cases with feedback (FPA) and without feedback. The four color lines illustrate the movement trajectory for the different directions. Gray spheres represent objects. (**A**) Real movement trajectory. (**B**) Predicted movement trajectory without feedback. (**C**) Final predicted movement trajectory with feedback (FPA). (**D**) Endpoint comparison. Blue spheres indicate endpoints of predicted movement trajectory and red spheres depict endpoints of compensated movement trajectory. Radii of blue and red spheres represent SDs of endpoints in cases with feedback and without feedback, respectively.

Fig. 4 shows the example results of one subject during one session. Predicted movements without feedback roughly followed the original movements (Figs. 4B and 4D). However, the predicted trajectory without feedback often did not reach the target. On the other hand, the predicted movements with feedback almost did reach the target (Figs. 4C, and 4D). Although real movements were somewhat scattered because the subject was instructed to move as fast as possible during the task, the predicted movement trajectory with feedback was more focused on the target because the predicted trajectory with feedback was compensated toward the target based on the target position. Improvement of prediction accuracy is more clearly represented in Video S1.

## Discussion

We proposed a BMI framework combining image processing with a novel prediction method, the FPA that generates feedback information and modifies movement prediction. The FPA predicts a target in every FPA process based on the neural activity, modifies the predicted direction toward the target and modulates the magnitude of the predicted vector to easily reach the target. Because the target is predicted in every FPA process, the subject can change a movement goal at any time. We demonstrated that combining feedback information for movement prediction considerably improves prediction accuracy. The proposed method will improve the performance of the arm-control BMI system not only for non-invasive but also for invasive neural signals. Therefore, the FPA will promote the development of a practical BMI system.

### Importance of feedback information

Feedback information is very important in movement control. To generate a reaching movement, three processes are required [10]. First, in the movement planning process, the movement needs to be planned to determine the movement direction and distance based on the sensory information about the object and hand locations. Second, in the process of inverse kinematic transformation, the joint angle trajectories of the shoulder and the elbow are determined to achieve the movement. Third, in the process of inverse dynamic transformation, the torque of the shoulder and elbow should be calculated based on the angle trajectories. The three processes are called sensorimotor transformations and are achieved based on the relationship between the joint angles of the arm and the location of the hand in space.

However, neural representations of the relationship may not exactly describe the real relationships because of structural differences or errors in the model’s parameters [10]. Therefore, this causes movement inaccuracies and it is difficult to predict a movement exactly without feedback information.

To overcome the inaccuracy, we suggested the BMI framework with the FPA. The predicted movements with feedback (FPA) almost did reach the target by modifying the direction and magnitude of the predicted movement vector, although the predicted trajectory without feedback does not reach the target (Fig. 4).

A recent study also proposed a BMI that combined target information [22]. The suggested study method predicts the target from neural activity before movement initiation in the first stage and combines the predicted target with the trajectory prediction in the second stage. Although the study is similar to our study in terms of combining position information for movement prediction, there are several limitations. In the previous study, the object positions were determined and fixed on the screen. Therefore, the method cannot be applied to control a neural prosthesis because the object positions are unknown in real life. This differs to our method, in which BMI obtains the object position from image processing. Moreover, the method predicts the target once before the movement and utilizes it during the trajectory prediction. This causes two main problems. Firstly, if the initial prediction is incorrect, then the target information will disrupt the subsequent prediction. Secondly, although the initial prediction is correct, the user cannot change the movement goal until one trial ends. In contrast, our proposed algorithm, FPA, predicts the target in every time step, therefore several incorrect predictions do not critically affect the trajectory prediction and the user can change the movement goal at any time (see Video S2). Lastly, information about the start and end times of the trials is required to predict the target and trajectory separately, which is inappropriate for practical BMI.

In other BMI studies, the target information was also used to assist the cursor control during the training periods for the adaptation of the subject to the system [6], [23] or to determine the parameters of a prediction model [24]. However, the method requires target information. As described above, the method also cannot be used in real life because the target will be changed in various situations.

### Feasibility of practical brain-machine interfaces

The proposed BMI framework with FPA will enable the practical BMI. First, the suggested algorithm improves prediction accuracy, as mentioned above. Second, it is applicable regardless of the object number or position because the FPA uses the positions obtained from the image and the image processing is not affected by the number or position of the object. Third, the subject can change a target at any time because the FPA selects a target based on neural activity and compensates the prediction in every time step (Figs. 1 and 2 Video S1). Last, the suggested method can also be applicable to any patients regardless of their disability type because it uses the additional information obtained from an external camera. Moreover, it may be possible to provide automatic grasping control signals using image information regardless of the various sizes and shapes of objects, without decoding the sophisticated finger movement. Therefore, the proposed BMI framework with FPA will promote the realization and commercialization of BMI.

### Limitations

The FPA is effective only if the movements are predictable from neural activity. Although the FPA improves prediction accuracy in most cases, it may not improve the performance when the movement prediction is extremely inaccurate because the algorithm compensates the movement based on the position of the target, which is predicted from neural activity. For this reason, subjects 2 and 9 show relatively little improvement, although the errors were significantly reduced. For the same reason, in case objects are very close to each other, performance improvements by the FPA may be decreased because it is difficult to predict the target from neural activity.

Another limitation is that the proposed method requires an external camera. Therefore, the adherence of a camera may be cumbersome. Nevertheless, it may be more convenient for the user, because it will innovatively improve the performance.

## Supporting Information

Data S1 **The ‘Data S1’ consists of epoched_MEG and epoched_acc.** The ‘epoched_MEG’ is the 68 channel MEG data on motor-related area of 25 trials. The ‘epoched_acc’ is an accelerometer data measured on the index finger. Each cell of the data corresponds to the different direction movement. The sequence of the data is channels, time, and trials.
(MAT)

Video S1 **Correct compensation with feedback (FPA).** In the movie, the FPA modified the predicted direction toward the target and modulated the magnitude of the predicted vector to easily reach the target. As a result, the predicted trajectory with feedback (FPA) reached the target accurately, although the predicted trajectory without feedback did not reach the target. In the movie, the green ball refers to the home position, the blue balls represent objects, the red ball refers to the predicted target from brain activity, the blue arrows describe the *a prior* predicted movement vectors without feedback, the black arrows are compensation vectors generated by the FPA, the red line represents the finally predicted vectors by the FPA, and the blue dotted line refers to the *a prior* predicted movement trajectory without feedback. The movie shows the sequence of the *a prior* prediction, selecting the target, generating artificial feedback vector, and final prediction by FPA.
(AVI)

Video S2 **Incorrect compensation with feedback (FPA).** In the movie, the predicted trajectory reached the target rapidly due to the effect of the weight value. Because the movement was predicted offline, the feedback of the compensated prediction was not presented to the subject. Therefore, the predicted movement was outside the target, because the subject may want to reach continuously. This caused incorrect compensation and the predicted trajectory pointed out another target. Although this is a result of incorrect compensation, it shows that the FPA could change the target at any time.
(AVI)

## References

[1] Georgopoulos AP, Kalaska JF, Caminiti R, Massey JT (1982) On the Relations between the Direction of Two-Dimensional Arm Movements and Cell Discharge in Primate Motor Cortex. Journal of Neuroscience 2: 1527–1537. 7143039 10.1523/JNEUROSCI.02-11-01527.1982 PMC6564361

[2] Georgopoulos AP, Kettner RE, Schwartz AB (1988) Primate Motor Cortex and Free Arm Movements to Visual Targets in 3-Dimensional Space. 2. Coding of the Direction of Movement by a Neuronal Population. Journal of Neuroscience 8: 2928–2937. 3411362 10.1523/JNEUROSCI.08-08-02928.1988 PMC6569382

[3] Wessberg J, Stambaugh CR, Kralik JD, Beck PD, Laubach M, et al (2000) Real-time prediction of hand trajectory by ensembles of cortical neurons in primates. Nature 408: 361–365. 11099043 10.1038/35042582

[4] Hochberg LR, Serruya MD, Friehs GM, Mukand JA, Saleh M, et al (2006) Neuronal ensemble control of prosthetic devices by a human with tetraplegia. Nature 442: 164–171. 16838014 10.1038/nature04970

[5] Bradberry TJ, Gentili RJ, Contreras-Vidal JL (2010) Reconstructing Three-Dimensional Hand Movements from Noninvasive Electroencephalographic Signals. Journal of Neuroscience 30: 3432–3437. 20203202 10.1523/JNEUROSCI.6107-09.2010 PMC6634107

[6] Velliste M, Perel S, Spalding MC, Whitford AS, Schwartz AB (2008) Cortical control of a prosthetic arm for self-feeding. Nature 453: 1098–1101. 18509337 10.1038/nature06996

[7] Hochberg LR, Bacher D, Jarosiewicz B, Masse NY, Simeral JD, et al (2012) Reach and grasp by people with tetraplegia using a neurally controlled robotic arm. Nature 485: 372–U121. 22596161 10.1038/nature11076 PMC3640850

[8] Collinger JL, Wodlinger B, Downey JE, Wang W, Tyler-Kabara EC, et al (2013) High-performance neuroprosthetic control by an individual with tetraplegia. Lancet 381: 557–564. 23253623 10.1016/S0140-6736(12)61816-9 PMC3641862

[9] Judy JW (2012) Neural Interfaces for Upper-Limb Prosthesis Control. Ieee Pulse 3: 57–60. 10.1109/MPUL.2011.2181026 22481748

[10] Kandel ER (2012) Principles of neural science. New York: McGraw-Hill. p.

[11] Haralick RM, Shapiro LG (1985) Image Segmentation Techniques. Computer Vision Graphics and Image Processing 29: 100–132.

[12] Yeom HG, Kim JS, Chung CK (2013) Estimation of the velocity and trajectory of three-dimensional reaching movements from non-invasive magnetoencephalography signals. J Neural Eng 10: 026006. 23428826 10.1088/1741-2560/10/2/026006

[13] Wu W, Black MJ, Mumford D, Gao Y, Bienenstock E, et al (2004) Modeling and decoding motor cortical activity using a switching Kalman filter. Ieee Transactions on Biomedical Engineering 51: 933–942. 15188861 10.1109/TBME.2004.826666

[14] Kim SP, Simeral JD, Hochberg LR, Donoghue JP, Black MJ (2008) Neural control of computer cursor velocity by decoding motor cortical spiking activity in humans with tetraplegia. J Neural Eng 5: 455–476. 19015583 10.1088/1741-2560/5/4/010 PMC2911243

[15] Kim SP, Simeral JD, Hochberg LR, Donoghue JP, Friehs GM, et al (2011) Point-and-click cursor control with an intracortical neural interface system by humans with tetraplegia. IEEE Trans Neural Syst Rehabil Eng 19: 193–203. 21278024 10.1109/TNSRE.2011.2107750 PMC3294291

[16] Thrun S, Burgard W, Fox D (2005) Probabilistic robotics. Cambridge, Mass.: MIT Press. xx, 647 p.

[17] Bradberry TJ, Rong F, Contreras-Vidal JL (2009) Decoding center-out hand velocity from MEG signals during visuomotor adaptation. Neuroimage 47: 1691–1700. 19539036 10.1016/j.neuroimage.2009.06.023

[18] Ganguly K, Secundo L, Ranade G, Orsborn A, Chang EF, et al (2009) Cortical Representation of Ipsilateral Arm Movements in Monkey and Man. Journal of Neuroscience 29: 12948–12956. 19828809 10.1523/JNEUROSCI.2471-09.2009 PMC3376707

[19] Flint RD, Lindberg EW, Jordan LR, Miller LE, Slutzky MW (2012) Accurate decoding of reaching movements from field potentials in the absence of spikes. J Neural Eng 9. 10.1088/1741-2560/9/4/046006 PMC3429374 22733013

[20] Hauschild M, Mulliken GH, Fineman I, Loeb GE, Andersen RA (2012) Cognitive signals for brain-machine interfaces in posterior parietal cortex include continuous 3D trajectory commands. Proc Natl Acad Sci U S A 109: 17075–17080. 23027946 10.1073/pnas.1215092109 PMC3479517

[21] MacKenzie IS, Kauppinen T, Silfverberg M (2001) Accuracy measures for evaluating computer pointing devices. Proceedings of the SIGCHI Conference on Human Factors in Computing Systems. Seattle, Washington, USA: ACM. 9–16.

[22] Shanechi MM, Williams ZM, Wornell GW, Hu RC, Powers M, et al.. (2013) A Real-Time Brain-Machine Interface Combining Motor Target and Trajectory Intent Using an Optimal Feedback Control Design. PLoS One 8. 10.1371/journal.pone.0059049 PMC3622681 23593130

[23] Fraser GW, Chase SM, Whitford A, Schwartz AB (2009) Control of a brain-computer interface without spike sorting. J Neural Eng 6. 10.1088/1741-2560/6/5/055004 19721186

[24] Mulliken GH, Musallam S, Andersen RA (2008) Decoding trajectories from posterior parietal cortex ensembles. Journal of Neuroscience 28: 12913–12926. 19036985 10.1523/JNEUROSCI.1463-08.2008 PMC2728059

